# Economic Evaluation of Clinical, Nutritional and Rehabilitation Interventions on Oropharyngeal Dysphagia after Stroke: A Systematic Review

**DOI:** 10.3390/nu15071714

**Published:** 2023-03-31

**Authors:** Sergio Marin, Omar Ortega, Mateu Serra-Prat, Ester Valls, Laia Pérez-Cordón, Pere Clavé

**Affiliations:** 1 Gastrointestinal Physiology Laboratory, Hospital de Mataró, Universitat Autònoma de Barcelona, 08304 Barcelona, Spain; 2 Pharmacy Department, Hospital Universitari Germans Trias i Pujol, 08916 Badalona, Spain; 3 Neurogastroenterology and Motility Research Group, Institut de Recerca Germans Trias I Pujol, 08916 Badalona, Spain; 4 Centro de Investigación Biomédica en Red de Enfermedades Hepáticas y Digestivas (CIBERehd), 08340 Mataró, Spain; 5 Research Unit, Hospital de Mataró, Consorci Sanitari del Maresme, 08304 Mataró, Spain; 6 Pharmacy Department, Hospital de Mataró, Consorci Sanitari del Maresme, 08304 Mataró, Spain

**Keywords:** deglutition disorders, nutrition therapy, stroke, brain ischemia/complications, cerebral haemorrhage, economics, health resources, systematic review

## Abstract

Background: Post-stroke oropharyngeal dysphagia (PS-OD) and its complications increase healthcare costs, suggesting that its appropriate management is cost-effective. We aimed to assess the efficiency of healthcare interventions in PS-OD management. Methods: A systematic review was conducted following PRISMA recommendations. Four databases were searched from inception through 30 June 2021. Outcome measures were cost-effectiveness and cost-savings of healthcare interventions. English and Spanish literature were included. Narrative and tables were used to present and synthesise evidence. Quality was evaluated using the CHEERS Statement. Results: A total of 244 studies were identified, and 10 were included. Screening and diagnosis of PS-OD studies found: (1) adjusted reduction in hospitalisation costs when assessed during the first admission day; (2) non-significant reduction in hospitalisation costs with OD management after thrombolysis; and (3) videofluoroscopy as the most cost-effective screening method (compared to bedside evaluation and a combination of both). Two studies showed cost-effective rehabilitation programmes, including OD management. Pelczarska et al. showed an incremental cost–utility ratio of texture-modified diets using a gum-based thickener of 20,977 PLN (4660€) following a dynamic model, and Kotecki et al. commercially prepared thickened fluids that were 44% to 59% less expensive than in situ prepared fluids. Elia et al. showed home enteral nutrition was cost-effective (£12,817/QALY), and Beavan et al. showed higher nutrient intake and low increase in hospitalisation costs using looped-nasogastric tubes (£5.20 for every 1% increase). Heterogeneity between studies precluded a quantitative synthesis. Conclusions: Included studies suggest that healthcare interventions aiming to prevent OD complications are cost-effective. However, studies assessing novel strategies are needed.

## 1. Introduction

Oropharyngeal dysphagia (OD) is one of the main complications suffered by post-stroke patients [1,2]. OD is highly prevalent in the acute stroke phase (40–78%), and although improvements can be observed during post-stroke recovery, it becomes a chronic condition in up to half of cases [1,3,4,5]. Post-stroke OD (PS-OD) impairs the efficacy and safety of swallowing, which causes severe complications such as dehydration, malnutrition, respiratory infection, aspiration pneumonia, hospital readmission and death [6,7,8,9]. PS-OD leads to a deterioration in the health status of patients, and it is an independent risk factor for longer hospital stay, institutionalisation after hospital discharge, poorer functional capacity and increased mortality 3 months after stroke [3]. Patients with PS-OD had longer hospital stays [10] and increased healthcare-related costs of around 15,000€ (approx. 16,900 USD), which could reach 24,000€ (approx. 27,600 USD) in patients with a pneumonia episode [11,12]. A recent study aiming to assess the independent cost of PS-OD and its main complications (malnutrition and respiratory infections) found that OD produced a significant and independent increase in healthcare costs of 789.68€ during hospitalisation and 873.50€ in the subacute stroke phase. Additionally, significant and independent costs were attributed to malnutrition and respiratory infections, two well-recognized complications of PS-OD [13]. In this study, the presence of poorer nutritional status (at risk of malnutrition or malnourished) led to a significant and independent increase of 1277.39€ during the sub-acute stroke phase (3-months follow up) and 2303.38€ in the chronic phase (12-months follow up); the presence of at least one episode of respiratory infection was associated with a significant and independent increase of 3792.62€ in the sub-acute stroke phase and 3034.08€ in the chronic phase; and a greater significant increase in healthcare-related costs at 12-months follow up was observed in those patients who had OD, were at risk of malnutrition or malnourished and had an episode of respiratory infection vs. those patients who did not develop PS-OD (19,817.58 ± 13,724.83 vs. 7242.80 ± 7402.60€, *p* < 0.0004) [13].

Appropriate management of PS-OD involves early screening and diagnosis of patients, compensation of the deglutition-impaired mechanisms and restoration of the swallowing function. This process is needed in order to avoid PS-OD-related complications and to improve patients’ health and quality of life [4,14]. The screening of OD is recommended by the AHA/ASA guidelines in the early stroke phases before the patients begin eating, drinking or taking oral medication [15] and is also recommended by the position statements of the European Society for Swallowing Disorders (ESSD) and by the recent European Guideline on diagnosis and treatment of PS-OD developed by the European Stroke Organisation (ESO) and the ESSD [16,17].

Current management of PS-OD is not standardised and can go from classical compensatory strategies to more innovative restorative strategies. Classical treatment for OD is mainly based on compensatory strategies to avoid biomechanical impairment related to the efficacy and safety of deglutition, such as the use of thickened fluids, texture-modified foods and postural changes [18]. There is evidence showing that viscosity and texture modification minimises the risk for aspiration during swallowing [19]. Several studies in PS-OD patients using thickened fluids during videofluoroscopy have shown that increasing shear-viscosity causes a strong viscosity-dependent therapeutic effect on the safety of swallowing, reducing laryngeal vestibule penetrations and tracheobronchial aspirations and their severity [18,20]. Changes in posture, such as chin down or head turn, usually change the biomechanics and pharyngeal and intrabolus pressure generation during swallowing and may reduce the incidence of aspiration in the specific cases [21,22]. 

On the other hand, innovative strategies that aim to restore the swallowing function are divided into peripheral sensory and central stimulation [23]. Peripheral stimulation includes the use of pharmacological agents such as transient receptor potential (TRP) channel agonists as well as intrapharyngeal or transcutaneous electrical stimulation (TES) strategies to increase the sensory input to improve the oropharyngeal swallow response. A recent systematic review on pharmacological agents for neurogenic dysphagia concluded that TRP channel agonists may be beneficial for neurogenic dysphagic patients [24]. Regarding TES, the National Institute for Health and Care Excellence (NICE) declared it as a safe and potentially beneficial therapy for PS-OD patients, although the actual evidence is limited in quality/quantity [25,26]. We previously found that TES greatly improved the safety of swallow and reduced the need for fluid thickening in PS-OD patients and that the effects were maintained in the long term [27].

Central stimulation strategies, including transcranial direct current stimulation (tDCS) and repetitive transcranial magnetic stimulation (rTMS), are currently being developed [28]. A recent review on non-invasive brain stimulation concluded that evidence suggests that rTMS and tDCS show promise as a treatment for PS-OD [29]. A systematic review and meta-analysis on the efficacy of noninvasive neurostimulation therapies for PS-OD patients found that rTMS, tDCS and electrical stimulation were effective for treating these patients; furthermore, rTMS was the most effective therapy [30]. Finally, a review on the current evidence on treatment for OD concluded that the quality of evidence that supports the use of innovative treatments is comparable if not better than classical treatments, as their clinical efficacy is evaluated by randomised controlled trials mainly in patients with PS-OD. However, there is still a high heterogeneity in treatment regimens, long-term effects, underlying mechanisms, and the effects of these strategies in other phenotypes of OD patients [31].

Although some important data on the healthcare-related costs associated with PS-OD and its main complications have emerged recently, the potential cost-savings and the cost-effectiveness of the mentioned strategies is not well known [12,13]. We hypothesise that these strategies not only can improve patients’ health status and quality of life, but can also provide important cost savings by avoiding the development of PS-OD complications. These interventions are safe, simple and based on the best scientific evidence collected over decades [30]. The purpose of this study is to collect all the available literature on the efficiency or cost-effectiveness of clinical, rehabilitation, and nutritional interventions such as early PS-OD screening and assessment as well as on the compensatory and neurorehabilitation strategies for the management of PS-OD.

## 2. Materials and Methods

A systematic review on the economic evaluations of clinical, rehabilitation and nutritional interventions on the appropriate management of PS-OD in patients who suffered a stroke was performed. This systematic review was performed following the recommendations stated by the Preferred Reporting Items for Systematic Reviews and Meta-analysis (PRISMA) [32]. The protocol for this systematic review was registered in the international prospective register of systematic reviews of the Center for Reviews and Dissemination (PROSPERO) (registration number: CRD42020136245) and published in a peer-reviewed journal [33]. The main outcome of interest was cost together with the health benefits (such as cost-effectiveness) associated with the healthcare interventions aimed to assess, treat and care for PS-OD. The processes to carry out this systematic review (literature selection process and extraction and quality assessment of included articles) together with task organisation and the resource and data management software used are also explained in the protocol of this study [33].

### 2.1. Literature Search

We searched MEDLINE using PubMed, Embase using Ovid, the National Health Service Economic Evaluation Database (NHS-EED) using the Center for Reviews and Dissemination Database of the University of York and the Cost-Effectiveness Analysis (CEA) Registry database of the Center for the Evaluation of Value and Risk in Health. English and Spanish literature was searched from inception through 30 June 2021. The systematic review did not include posters, abstracts, book chapters or unpublished literature. The search strategy (combined MeSH and search terms used) is available in the previously published protocol of this study and reported in the Supplementary Material [33].

### 2.2. Selection Process

Studies identified through literature search were selected using a double-phase process. In the first phase, a screening of the title and abstract of the identified articles was performed to avoid studies not reporting at least minimal relevant information in this field. A second phase to exclude articles according to eligibility criteria was performed. Studies were included if they (i) had economic evaluations in which the intervention effect was quantified by effectiveness results or other measures of effect on healthcare (cost minimization, cost-utility, cost-effectiveness and cost–benefit analysis) or (ii) studies in which cost-savings-applying healthcare interventions in OD management were assessed (studies in which potential savings were reported by applying interventions on OD assessment and management) and provided information on post-stroke adult patients (≥18 years) with PS-OD. Studies were excluded if they were (i) partial economic evaluation studies such as cost-of-illness studies, (ii) studies on oesophageal dysphagia management, or (iii) studies in which OD was related to an aetiology different than stroke or (iv) duplicate publications of the same study.

### 2.3. Data Presentation and Summary Measures

Data were reported in their original format using tables and narrative. A meta-narrative method and tables were used to synthesise evidence. Results are presented in the following order: (i) studies reporting economic evaluations of the screening and/or assessment of OD, (ii) studies reporting economic evaluations of rehabilitation programs that included interventions on PS-OD, (iii) economic evaluation studies of OD interventions related to compensatory treatment strategies such as food consistency modification and, (iv) studies reporting economic evaluations of enteral tube feeding nutrition and provision in patients with PS-OD. Information was presented according to the Centre for Reviews and Dissemination recommendations [34]. Finally, a meta-narrative synthesis of the extracted information was performed in which we described both the assessed evidence on the efficiency of different interventions together with some of the key aspects of quality assessment evaluation. We reported whether studies identified, measured, and assessed the complete form of all the important costs for each assessed alternative, whether the study structure (study approach, data source) was performed in the most appropriate way to answer the study question and whether the most important factors to understand these economic evaluations were properly reported.

### 2.4. Quality Assessment

A specific tool to assess the internal validity and the reporting of key factors of economic evaluation studies was used. We applied the Consolidated Health Economic Evaluation Reporting Standards 2022 (CHEERS 2022) Statement [35]. A set of items that apply to a critical appraisal of economic evaluation studies is provided in this checklist. Each item represents a study aspect that we rated as “Yes, partly, no, or not applicable”. For each study, the total amounts of items rated as “yes (1 point)” and “partly (0.5 points)” were divided between the total applicable items. This was expressed as a percentage; a higher score represents a lower risk of bias. We consider a score of 100% as a study with very low risk of bias. Of note, this specific tool does not provide an overall score of the internal validity of the assessed studies (including outcomes and aims different from economic evaluations), only of the economic-related outcomes and aims. As we wanted to assess the current state of the literature on this topic, and the heterogeneity across included studies precluded a final quantitative synthesis of the evidence, we did not exclude any study from this review based on its quality assessment score nor the directness in relation to this review question.

## 3. Results

**Figure 1:**
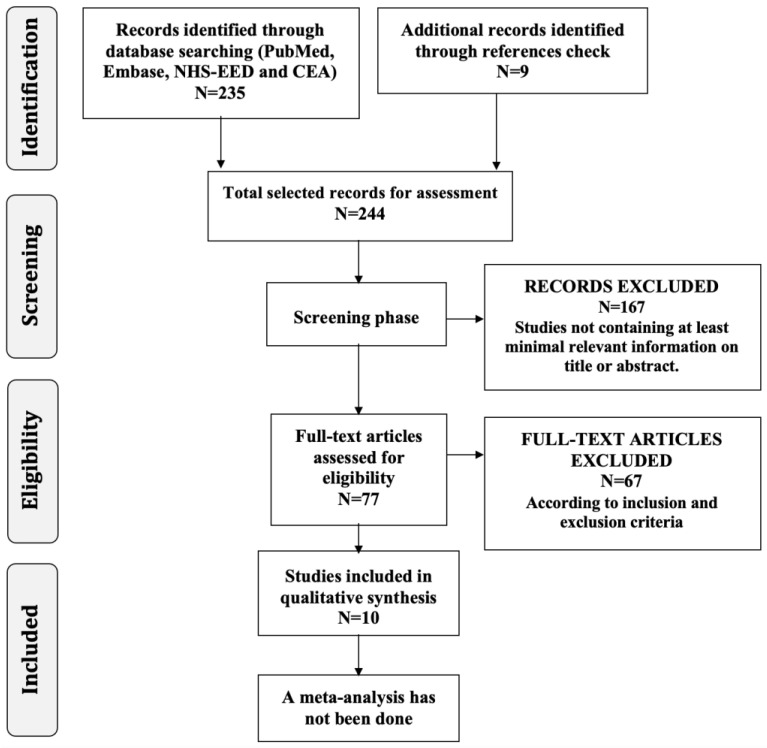
Selection process flow diagram.

A total of 235 articles were identified in the bibliographic database search (134 through MEDLINE using PubMed, 53 through Embase using Ovid, 42 through NHS-EED and 6 through the CEA Registry database), and 9 additional articles were identified through a bibliographic reference check. A total of 244 studies were assessed in the selection phases. After screening both the title and abstract of these articles, 167 articles were excluded in the first selection phase because they did not provide at least minimal relevant information on the topic. A second evaluation phase was carried out with full text review of the 77 remaining studies. After this second evaluation phase, 67 articles were excluded because they did not meet the criteria for inclusion (44 were not economic evaluation studies, 10 were partial economic evaluation studies, 6 did not refer to OD, in 1 study OD was not related to stroke, 5 were duplicated articles, and 1 did not fulfil the publication criteria as it was an abstract); finally, 10 articles were included in this systematic review (Figure 1) [36,37,38,39,40,41,42,43,44,45].

The data and features of the included studies were summed up and presented both in a narrative presentation and evidence tables. Table 1 summarises the main design characteristics of the studies. Table 2 summarises specific economic characteristics of the included studies, such as the elements of cost considered and the used currency. Table 3 summarises characteristics of study populations. Table 4 summarises the results of individual studies along with quality evaluation scores. Study designs, economic evaluation methods and techniques, study interventions, outcome measurements, cost elements considered, settings, and perspective were heterogeneous across the studies, which precluded a quantitative synthesis.

### 3.1. Data Presentation and Results of Individual Studies

#### 3.1.1. Screening and Assessment of PS-OD

Four studies assessed different strategies to assess PS-OD in hospitalised patients with acute stroke, from different perspectives and using different methodologies. Three of these studies had a longitudinal design, and one was a model-based study. Study sample size ranged from 83 to 5909 adult (≥18 year) participants [36,37,38,39]. Each study was performed in a different country: China [36], Denmark [37], the United States [38], and Australia [39]. The Chinese, Australian and Danish studies evaluated cost-savings [36,37,39], and the study performed in the United States was a cost-effectiveness study [38].

Liu, Z.Y. et al. analysed the differences in total hospitalisation costs, comparing a period when the water-swallowing test (WST) was systematically performed on acute ischemic stroke patients and a posterior period when all patients underwent the WST, and the volume-viscosity swallow test (V-VST) was performed on those who failed the WST. This study did not find differences in the total hospitalisation costs nor in length of hospitalisation between patients assessed with the WST and those assessed with the WST and the V-VST if required. Median total hospitalisation costs were 2907.80 USD for patients assessed with the WST and 2899.40 USD for those patients who were assessed with the WST and then the V-VST if required. However, this study found a significant and relevant reduction in the occurrence of pneumonia (21.8% vs. 10.5%, *p* = 0.024) and in the rate of nasogastric tube feeding (25.9% vs. 14.7%, *p* = 0.040) when the V-VST was systematically applied [36].

Schwarz, M. et al. assessed the impact of an OD screening protocol in which OD was assessed by a speech-language pathologist in thrombolysed ischemic stroke patients on the operational outcomes (hospitalisation cost, length of stay, and sanitary service compliance) from the hospital perspective. This study found a non-significant reduction in hospital costs of 1505 Australian dollars (18,053 vs. 16,548 Australian dollars) when a clinical protocol aimed at managing dysphagia in post-stroke thrombolysed patients was utilized [39]. 

Svendsen, M.L. et al. evaluated the adjusted reduction in hospitalisation costs when a set of healthcare processes, such as swallow assessment through Gugging Swallow Screen (GUS), were performed early after acute stroke admission. To do this, this study compared total direct sanitary costs of hospitalisation based on stays in different hospital units, comparing patients in whom the swallowing function was assessed early after hospital admission and those who were not. This study found an adjusted reduction in hospitalisation costs in patients in whom the swallowing function was assessed during the first day of hospitalisation after acute stroke of 12,556 USD (95% CI 9751–15 361) after adjusting for a set of confounders such as age or modified Rankin Scale and Charlson comorbidity scale scores [37].

Wilson, R.D. and Howe, E.C. compared the cost-effectiveness of different screening methods for the detection of OD in hospitalised stroke patients (videofluoroscopic swallow assessment vs. clinical bedside swallow assessment vs. a combined approach in which videofluoroscopy was performed on patients with abnormal swallows at bedside assessment). This study was performed by using a decision-analysis model created with data from multiple sources. Direct medical costs of videofluoroscopy, non-oral feeding and pneumonia were considered. Quality-adjusted life-years (QALY) were measured, and an incremental cost-effectiveness analysis was made. The time horizon was hospitalisation, and analysis was performed from the societal perspective. This study found that videofluoroscopy was a more effective and cheaper screening method for OD in patients with acute stroke when compared with clinical bedside swallow assessment alone or a combination of both (QALY/costs: videofluoroscopy: 1.791 QALYs/1853 USD; clinical bedside swallow evaluation: 1.789 QALYs/1968 USD; and combined approach: 1.790 QALYs/1943 USD) [38]. 

#### 3.1.2. Rehabilitation Services including PS-OD Management

Two studies assessed different rehabilitation programmes that included hospitalised patients with stroke, from different perspectives and using different methodologies [40,41]. These two studies were longitudinally designed with prospective data gathering [40,41]. Study sample sizes were 207 [40] and 50 [41]. Both studies were performed in Thailand [40,41]. One was a cost-utility analysis [28], and the other a cost-effectiveness analysis [41].

Khiaocharoen, O. et al. analysed the cost-utility of a rehabilitation programme that included swallow training (together with physical therapies such as exercise, trunk and walking training and occupational therapies such as self-care, cognitive and communication training) in subacute and non-acute hospitalised post-stroke patients and after discharge in two regional hospitals in Thailand. Swallow training consisted of orofacial motor skills training and swallowing and eating therapy included in a set of occupational therapy activities. To achieve this, the study measured patients’ functional status according to a modified Barthel index and quality of life following the EuroQol five-dimensional questionnaire (and converted to a utility score). This study analysed direct healthcare costs such as rehabilitation and medical costs, direct non-healthcare costs such as the costs of living during the stroke episode (e.g., transport or food) and indirect costs such as patients’ and relatives’ loss of income during hospitalisation and up to 4-months’ follow-up from the societal perspective. This study found a significantly higher average gained utility score for the group of patients that received those rehabilitation services more than once compared with those who did not receive them or just once (0.632 vs. 0.352). Moreover, the incremental cost per QALY for those patients who received rehabilitation services was 24,571 bahts (incremental cost-effectiveness ratio) when costs were assessed from a societal perspective (and 19,971 bahts when assessed from a governmental one) and that this ratio was robust in terms of program costs and outcome uncertainty estimations [40].

Suksathien, R. et al. analysed the outcomes and cost-effectiveness of a short-course inpatient rehabilitation program that included speech-language therapy (together with physical and occupational therapy) in a tertiary hospital in Thailand. This study measured mean patient functional status differences between admission and discharge according to the Barthel index, as well as mean total costs by assessing direct healthcare costs such as the costs of medicine, laboratory time, rehabilitation training and nursing from the hospital perspective. This study found a mean change in the Barthel index score between discharge and admission of 5 ± 2.25 (95% CI 2–10) and mean total costs of 7729 ± 4330 (95% CI 1828–22,450) baht (incremental cost per each one-point change in the Barthel index score of 1545.8 bahts). Of note, only 10% of included participants suffered OD, and a specific assessment of OD evolution across rehabilitation programme performance was not reported [41].

#### 3.1.3. Compensatory Strategies: Food Consistency Modification and Thickened Fluids

Two studies assessed interventions on food consistency modification therapies [42,43]. One study was a cost-savings analysis [42], and the other a model-based cost-utility analysis [43]. One study was performed in the United States [42], and the other in Poland [43].

Kotecki, S. and Schmidt, R. compared the cost of thickened liquids prepared by nursing staff using Resource ThickenUp^®^ with the cost of using commercially prepared thickened liquids. To do this, the study analysed how experienced hospital nurses and technicians prepared nectar- and honey-consistency thickened water, milk, and orange juice. This study was performed in a neuroscience acute care unit of a large urban community hospital. This study analysed the time spent and the cost of the compared interventions: the use of thickened liquids to the consistency of nectar and honey from water, milk and orange juice prepared by nurses using Resource ThickenUp^®^ vs. the use of commercially prepared thickened liquids. Costs were measured from the hospital perspective, and the time horizon was hospitalisation. This study found commercially prepared thickened liquids were 44% to 59% less expensive than those manually prepared by nurses and technicians [42].

Following a different methodology, Pelckzarska, A. et al. assessed the cost-utility of texture modified foods with xanthan-gum-based Nutilis Clear^®^ compared with routine clinical practice (behavioural compensations, manoeuvres, and rehabilitation exercises). To do this, the study followed two different models: (i) an 8-week fixed simple-equation static model in which the duration of OD was fixed and (ii) a 1-year Markov dynamic model in which the duration of OD was modelled. This study was limited to persons who tolerate semisolid intake but not fluids (score 10 to 14 according to GUS) and analysed the costs, the health state utilities, and clinical events such as aspiration, aspiration pneumonia and death. Analysed costs were direct OD treatment costs, aspiration pneumonia treatment costs and monitoring costs (only in the dynamic model) and were assessed from the public payer perspective. This study found cost-effective food consistency modification therapy with xanthan-gum-based Nutilis Clear^®^ (incremental cost-utility ratio of 21,387 PLN and 20,977 PLN following a static and dynamic model, respectively). Moreover, these findings were confirmed following one-way, multi-way and probabilistic sensitivity analysis. This study also found the cost of xanthan-gum-based products as the major contributor to costs, the reduction of aspiration pneumonia prevalence as the major contributor to costs-savings and the utility increment of aspiration avoidance and the risk of aspiration pneumonia occurrence as the factors with a major impact on study results, according to sensitivity analyses [43]. 

#### 3.1.4. Nutrition by Enteral Tube Feeding in Patients with PS-OD

Two studies reported costs associated with health interventions related to the use of tube feeding in patients affected by cerebrovascular diseases [44,45]. One of these studies had an observational, longitudinal design with a sample size of 9895 patients (25 patients for the quality-of-life assessment) [44], and the other was a randomised clinical trial with a sample size of 104 patients [45]. Both were performed in the United Kingdom [44,45]. One study reported costs savings associated with looped nasogastric tube feeding use [45], and the other was a cost-utility study [44].

Beavan, J. et al. analysed the cost-effectiveness of nourishing acute stroke patients using a looped nasogastric tube compared to the use of a nasogastric tube secured by an adhesive nasal sticker. This study analysed the additional cost associated with any 1% of additional nutrition received of the total nutrition prescribed using the strategy of the looped nasogastric tube. Feeding costs were assessed over 2 weeks in acute stroke patients. This randomised clinical trial found that patients assigned to the looped nasogastric tube received a higher mean volume of feed and fluids (17% more, 95% CI 5% to 28%) and estimated that the incremental cost per any 1% additional total nutrition prescribed was 5.20 sterling (GBP) (loop group, mean costs: 426 GBP vs. control group, mean costs: 338 GBP) [45].

Elia, M. and Stratton, R.J. analysed the cost-utility of the application of enteral tube feeding in patients who had suffered a cerebrovascular accident. This study assessed in-hospital costs such as costs of gastrostomy insertion, patient training and readmissions, the cost of nursing homes and costs beyond acute hospitalisation such as home visits by general practitioners, dietitians, nurses, speech and language therapists, physiotherapists, chiropodists, community occupational therapists and the cost of feeding, including ancillaries and delivery, in 9895 patients who initiated enteral tube feeding beyond hospitalisation from 1995 to 2005. QALYs were assessed on a sub-sample of 25 participants for 3 years, and the cost per each gained QALY was calculated and quality of life measured following the EuroQol visual analogue scale. This study found that the cost per QALY for those patients who received enteral tube feeding at home was £12,817 (95% CI for quality of life £10,351–£16,826) and that this ratio was insensitive to the frequency of received home visits, the outcome of patients who reversed to full oral feeding, and the computed outcome of a “control” group not given enteral tube feeding. In the case of patients who received enteral tube feeding nutrition in nursing homes, the cost per QALY varied from £10,303 to £68,064. The value of cost per QALY in this wide range on the incremental cost-utility ratio of enteral tube feeding nutrition in those patients in nursing home depended on the state contribution to non-medical costs [44].

### 3.2. Synthesis of the Studies Findings

#### 3.2.1. Screening and Assessment of PS-OD

For acute stroke patients who were assessed with the WST vs. the WST and the V-VST if the first failed, Liu, Z.Y. et al. did not find significant differences in total hospitalisation costs in China though significant and relevant reductions in pneumonia occurrence and in the rate of nasogastric tube feeding when the V-VST was systematically used [36]. A non-significant reduction of 1505 Australian dollars in hospital costs during admission was found when assessing the implementation of a protocol aimed to manage OD in post-stroke thrombolysed hospitalised patients [39]. However, an independent reduction in hospitalisation costs in Denmark was observed in patients in whom swallowing function was assessed early after acute stroke (first 24 h) compared to those in whom it was delayed [37]. In the case of screening methods for OD after acute stroke admission, one study showed videofluoroscopy as the most cost-effective approach when compared with clinical bedside swallow evaluation or a combined approach (1034 USD/QALY) in the United States [38].

#### 3.2.2. Rehabilitation Services including PS-OD Management

Rehabilitation services that included post-stroke inpatient swallow training (starting at the subacute and non-acute stroke phases) was found to be cost-effective from the societal perspective in Thailand (incremental cost-effectiveness ratio of 24,571 bahts per QALY gained, below the established threshold of 100,000 bahts per QALY, or about 2780 euros) [40]. Moreover, a short-course in-hospital rehabilitation program that included speech-language therapy saw a favourable mean change in the Barthel index score of 5 points between discharge and admission, with a mean total cost of 7729 bahts [41].

#### 3.2.3. Compensatory Strategies: Food Consistency Modification and Thickened Fluids

The use of texture-modified diets using a gum-based thickener was found to be cost-effective from the public payer perspective in Poland (incremental cost-utility ratios from 21,387 PLN to 20,977 PLN per QALY gained, or about 4750–4660 euros, below the established threshold of 147,024 PLN per QALY in Poland) following a two-model-based approach and confirmed through comprehensive sensitivity analyses [43]. Moreover, the only study that assessed this point found commercially prepared thickened liquids more consistent regarding low viscosity variability and less expensive compared with those prepared in situ (44% to 59%) from the hospital perspective in the United States [42].

#### 3.2.4. Nutrition by Enteral Tube Feeding in Patients with PS-OD

The use of enteral tube feeding nutrition at home was found to be cost-effective in the United Kingdom (£12,817 per QALY gained and from £10,351 to £16,826 using the 95% CI of quality-of-life measurements, below the established threshold of £30,000 per QALY). However, in the case of patients attended to in nursing homes, the incremental cost-effectiveness ratio depended on the state contribution to non-medical costs and varied from £10,303 to £68,064, with the lower value of this range corresponding to when the state did not contribute to overall costs and the higher when the state contributed 100% of overall costs (e.g., less than £30,000 per QALY gained with a state contribution less than 34%) [44]. Moreover, a study performed in the United Kingdom reported that those patients receiving feeding through looped nasogastric tubes received a 17% higher mean volume of feed and fluids and estimated £5.20 as the cost of receiving the 1% additional total nutrition prescribed using this strategy [45].

### 3.3. Quality Assessment

Risk of bias was assessed for each study. Total calculated scores for each study are presented in Table 4 along with the main results of presented studies, and specific evaluations for each item are available in Table S1 of the Supplementary Materials. Scores ranged from 54.1% to 83.9%. The mean quality studies score was 72.1%. Only one study assessed costs other than direct healthcare costs, such as direct non-healthcare costs or indirect costs [40]. Only two studies offered analysis performed from the societal perspective [38,40]. Only one study described the use of a discount rate to obtain an estimation of the present value of costs [44]. Sensitivity analyses to assess the robustness of the performed investigations were performed in three studies [40,43,44]. Target population and subgroups, comparators, health outcome measurements and cost and resource estimations were generally widely described in the included studies.

## 4. Discussion

This systematic review provides a comprehensive description of the available literature on the efficiency of some healthcare interventions to detect and manage PS-OD. Economic evaluation and cost-savings studies on healthcare interventions in the detection of PS-OD, rehabilitation programmes in which OD was considered, and the use of food-consistency modification strategies and tube-feeding nutrition were presented and summarized [36,37,38,39,40,41,42,43,44,45].

Regarding the efficiency of screening and diagnosis of PS-OD, Liu, Z.Y. et al. did not find differences in hospitalisation costs when PS-OD was assessed with the WST vs. V-VST if the WST failed, although this study did find a significant and relevant reduction in pneumonia incidence and also in the rate of nasogastric tube feeding in those patients assessed with the V-VST, providing new data on the clinical utility of the V-VST, which has been recently confirmed by comprehensive studies [36,46]. A recent systematic and scoping review on the psychometrics and clinical utility of the V-VST in the clinical screening and assessment of OD also developed by our group found V-VST had a diagnostic sensitivity for OD of 93.17%, a specificity of 81.39%, and an inter-rater reliability Kappa of 0.77 and concluded that V-VST has strong psychometric properties and valid endpoints for OD in several phenotypes of patients, including PS-OD. Our results support its utility in the screening and clinical diagnosis and management of OD [46]. On the other hand, Schwartz, M. et al. found a non-significant reduction of 1505 Australian dollars in hospitalisation costs using a protocol to manage OD after thrombolysis [39]. Of note, the prospective study performed by Svendsen, M.L. et al. in Denmark found an adjusted reduction in hospitalisation costs of more than 12,500 USD when swallow function was assessed during the first 24 h after acute stroke admission, emphasising the importance of early swallow assessment not only from a clinical perspective but also from an economic one [37]. Finally, Wilson, R.D. and Howe, E.C. found a videofluoroscopic swallowing study more cost-effective than clinical bedside swallow evaluation or a combined approach through model-based decision-analysis [38]. We believe that programs including systematic early screening and assessment of PS-OD are clinically profitable, as they reduce the nutritional and respiratory complications of PS-OD and improve the clinical outcomes of PS-OD patients and also reduce costs. Moreover, the use of innovative strategies such as artificial intelligence, which can provide accurate and systematic universal screening for OD to hospitalised patients, could improve the cost-effectiveness of OD assessment and further reduce complications and hospitalisation costs [47].

Only two economic evaluation studies are available in the literature regarding OD compensatory-treatment strategies. The study performed by Pelckzarska, A. et al. is the first economic evaluation study addressing the cost-effectiveness of food-consistency modification compared with routine clinical practice. This study showed the cost-effectiveness of food consistency modification with xanthan gum-based Nutilis Clear^®^ in the treatment of PS-OD in Poland from the public payer perspective [43]. The model-based cost-savings analysis performed by Kotecki, S. and Schmidt, R. found that the use of commercially pre-thickened fluids was less expensive than in situ preparation. Although both studies were promoted by the medical nutrition industry, they clearly show the cost-effectiveness of this compensatory therapeutic strategy. However, patients affected by severe and chronic PS-OD may require tube-feeding nutrition to avoid aspiration pneumonia and malnutrition [42]. Elia, M. and Stratton, R.J. analysed the cost-utility of enteral tube feeding beyond acute hospitalisation after cerebrovascular disease in the United Kingdom. This study showed the cost-effectiveness of enteral tube feeding at home and in nursing homes in which non-medical costs were paid privately. However, when the totality of the non-medical costs was paid by the state, the incremental cost-effectiveness ratio went up to £68,064/QALY, well above the established threshold of £30,000/QALY [44]. The measurement of QALYs and the assessment of the paid cost per incremental QALY (incremental cost-utility ratio) in studies assessing compensatory or enteral tube feeding strategies is necessary due to the nature of OD, which can affect not only patient mortality but also quality of life. Future economic studies assessing other interventions such as restorative ones need to be designed and carried out [26]. We recently developed a minimal-massive intervention (MMI) in hospitalised older patients with OD. This MMI consists of early screening of OD and of: (i) fluid thickening and texture-modified foods, (ii) caloric and protein supplementation; and (iii) oral health and hygiene recommendations during hospitalisation and following discharge. This intervention was recently improved by adding oral nutritional supplements in patients at risk of malnutrition. Our results suggest that MMI in hospitalised older patients with OD improves nutritional status and functionality and reduces hospital readmissions, respiratory infections and mortality. MMI might become a simple and cost-effective strategy to avoid OD complications in the geriatric population admitted with an acute disease to a general hospital, including those with PS-OD [48].

In recent years, the aim of post-stroke management of OD has been to restore the swallowing function using different strategies. In the present review, two studies assessed costs associated with rehabilitation services that included swallow training [40,41]. Khiaocharoen, O. et al. assessed the cost-utility of a rehabilitation programme including swallow training starting at the subacute and non-acute phases of stroke in Thailand and found the program cost-effective from the societal perspective in Thailand at a cost of 24,571 bahts per QALY gained (well below the 100,000 bahts/QALY established threshold in Thailand in the period which the study was carried out, 2008–2009) [40,49]. A subsequent study performed by Suksathien, R. et al. to assess outcomes of a short-course inpatient rehabilitation program that included speech-language therapy in Thailand reported a functional ability improvement on discharge with low costs. Of note, only 10% of participants included in this study suffered PS-OD, and non-specific OD-related outcome measures were used, which should be considered when interpreting the contributions of this last study for the specific aim of this systematic review [41].

The recently published ESO and ESSD guideline for the diagnosis and treatment of PS-OD found low to moderate quality of evidence for a variety of treatment options to improve swallowing physiology and swallowing safety. These options include dietary interventions, behavioural swallowing treatment including acupuncture, nutritional interventions, oral health care, different pharmacological agents and different types of neurostimulation treatment. Some of the studied interventions also had an impact on other clinical endpoints, such as feeding status or pneumonia [16]. This systematic review only partially fulfils the initially proposed objectives. The studies found assessed different health interventions and were conducted with very different methodologies regarding clinical and economic assessment. This limits the robustness of findings and reduces the synthesis to a narrative explanation of what has been reported to date. Heterogeneity between economic techniques used, assessed interventions, and cost elements considered precluded a quantitative synthesis and a meta-analysis. Moreover, no literature was found assessing the cost-effectiveness of restorative strategies, and data on the cost-effectiveness of OD management beyond acute stroke hospital admission is scarce. Despite this, this systematic review shows that some healthcare interventions on the detection and management of PS-OD have proven to be cost-effective or save costs in different settings. 

PS-OD is a dynamic condition and, although improvements can be observed during the first weeks of rehabilitation, it remains as a chronic condition in up to half of patients, and new signs and symptoms can arise in long-term follow up of patients with poorer functionality [5,50]. Early and comprehensive management of PS-OD is imperative during acute stroke hospitalization and subsequent rehabilitation phases to prevent severe clinical complications such as respiratory infections and malnutrition and to avoid the high healthcare costs of these complications [13]. In this context, the systematic detection and specialised management of PS-OD and the prevention of its complications could provide a cost-effective improvement of patient outcomes. A systematic summary of the literature on the efficiency and cost savings associated with comprehensive PS-OD management could help decision making in terms of the appropriate management and treatment of this condition and also bolster future research in this field to create an economic model that considers the full management of this disease.

Future studies must be performed on the evaluation of the cost-effectiveness of health interventions on the management of OD beyond acute hospitalisation and on the efficiency of restorative strategies. Future studies are needed to establish the cost-effectiveness of the appropriate management of PS-OD and its complications (malnutrition, dehydration and respiratory infections) not only during hospitalisation but also at the sub-acute and long-term phases of OD due its chronic impact. These studies could be a first step to create an economic model of the disease that could help healthcare providers with decision making.

## 5. Conclusions

This study has three main conclusions: (i) interventions in the early detection or management of OD that have a positive effect preventing the main complications of OD (malnutrition and respiratory infections) tend to be cost-effective by reducing hospitalisation costs and improving clinical outcomes; (ii) the cost-effectiveness of interventions beyond acute hospital admission has been little explored, and future studies are needed to assess the cost-effectiveness of appropriate management of OD after acute stroke hospitalisation; and (iii) in contrast to compensatory strategies, we found little evidence assessing the cost-effectiveness of strategies aimed at restoring the swallowing function after stroke. Studies assessing the cost-effectiveness of novel screening, assessment, compensatory and restorative strategies are needed.

## Figures and Tables

**Table 1:** Main design characteristics.

Study ID	Aim	Evaluated Intervention	Study Population	Design/Data Gathering	Time Horizon and Perspective
**Assessment of OD after Stroke**					
**Wilson, R,D.;** **2012** [38]	Cost-effectiveness of dysphagia screening methods	- VFS - CBSE - CBSE followed by VFS if swallow abnormal	Hospitalised acute stroke (model-based)	Cost-effectiveness Decision-analysis model NA	Hospitalisation Societal perspective
**Svendsen, M.L.;** **2014** [37]	Association between processes of early stroke care and hospital costs	Early assessment of the swallowing function and 10 other processes related to acute stroke care	Ischemic and haemorrhagic stroke patients ≥18 years	Cost-saving Prospective	Hospitalisation Hospital perspective
**Schwarz, M.;** **2017** [39]	Impact of an OD screening protocol for thrombolysed stroke patients on operational outcomes (cost, length of stay, service compliance)	Protocol for the early screening of OD in thrombolysed patients	Ischemic stroke ≥18 years	Cost-saving Retrospective	Hospitalisation Hospital perspective
**Liu, Z.Y.;** **2020** [36]	Differences in total hospitalisation costs assessing OD with the WST test vs. the WST test and the V-VST if the first failed	Early assessment of PS-OD with two different screening tests: - WST - WST followed by V-VST if the first failed	Ischemic stroke >18 years	Cost-saving Retrospective	Hospitalisation Hospital perspective
**Rehabilitation Services Including OD Management**					
**Khiaocharoen, O.;** **2012 [40]**	Cost-utility of rehabilitation programme, including swallow training	- Rehabilitation services (including swallow training) at subacute and non-acute phases - Comparator group that did not receive rehabilitation or just once	Hospitalised acute stroke (subacute and non-acute phases) and discharged >17 years	Cost-utility Prospective	4 months Societal perspective (also provided data on governmental)
**Suksathien, R.;** **2015 [41]**	Efficiency and cost of short-course rehabilitation program	Short-course rehabilitation program (including speech-language therapy)	Inpatient rehabilitation after stroke >18 years	Cost-effectiveness Prospective	Inpatient rehabilitation Hospital perspective
**Nutrition by Enteral Tube Feeding**					
**Elia, M.;** **2008 [44]**	Cost-utility of long-term enteral tube feeding	Enteral tube feeding	Cerebrovascular disease, nourished by enteral tube at home or nursing home	Cost-utility Retrospective	3 years after starting tube feeding NA
**Beavan, J.;** **2010 [45]**	Cost-effectiveness of nasal loop	Looped vs. non-looped nasogastric tube feeding	Hospitalised acute stroke	Cost-effectiveness Prospective (randomised controlled trial)	2 weeks Cost associated to feeding perspective
**Compensatory Strategies: Food Consistency Modification and Thickened Fluids**					
**Kotecki, S.;** **2010 [42]**	Differences in time and costs between nursing-staff-prepared and commercially prepared thickened liquids	Nursing-staff-prepared thickened liquids using Resource ThickenUp^®^ and commercially prepared thickened liquids	NA	Cost-saving NA	Hospitalisation Hospital Perspective
**Pelczarska, A.;** **2020 [43]**	Cost-utility of xanthan gum-based consistency modification therapy (Nutilis Clear^®^)	Routine clinical practice: behavioural compensations, manoeuvres, and rehabilitation exercises Xanthan gum–based consistency modification therapy (Nutilis Clear^®^)	Adult post-stroke (model-based)	Model-based cost-utility analysis (static and dynamic model) NA	Time horizon: -Static model: 8 weeks -Dynamic model: 1 year Public payer perspective

Abbreviations: CBSE: clinical bedside swallowing evaluation; ID: identification; OD: oropharyngeal dysphagia; NA: not available/not applicable; PLN: polish zloty; VFS: videofluoroscopy; V-VST: volume-viscosity swallow test; WST: water swallowing test.

**Table 2:** Specific characteristics of included studies: data source and elements of cost considered.

Study ID	Data Source	Elements of Cost Considered: (a) Direct Healthcare Costs (b) Direct Non-Healthcare Costs (c) Indirect Costs	Country, Year and Currency
**Assessment of OD after Stroke**			
**Wilson, R.D.;** **2012** [38]	Available literature Estimations	(a) Yes, direct medical costs of VFS, non-oral feeding and pneumonia (b) No (c) No	United States NA USD (2010)
**Svendsen, M.L.;** **2014 [37]**	Medical registries, national population-based, including the Danish Stroke Registry, the Danish National Registry of Patients, and the Danish Civil Registration System	(a) Yes, hospitalisation costs (b) No (c) No	Denmark 2005–2010 USD (2010)
**Schwarz, M.;** **2017** [39]	Clinical records	(a) Yes, hospitalisation costs (b) No (c) No	Australia 2011–2014 Australian dollars (year not available)
**Liu, Z.Y.;** **2020** [36]	Questionnaire Clinical records	(a) Yes, hospitalisation costs (b) No (c) No	China 2017 USD (year not available)
**Rehabilitation Services Including OD Management**			
**Khiaocharoen, O.;** **2012 [40]**	Data collection process by health professionals and cost diary recorded by patients and relatives both checked and confirmed by investigators	(a) Yes, including rehabilitation and medical costs (b) Yes, cost of living during the stroke episode (e.g., transport, food) (c) Yes, loss of income of patients and relatives	Thailand 2008–2009 Baht (year NA)
**Suksathien, R.;** **2015 [41]**	Data collection process by investigators	(a) Yes, medicine, laboratory, rehabilitation training, nursing, bed, and others (not defined) (b) No (c) No	Thailand 2014 Baht (year NA)
**Nutrition by Enteral Tube Feeding**			
**Elia, M.;** **2008 [44]**	Database of the British Artificial Nutrition Survey	(a) Yes, in hospital cost of gastrostomy insertion and patient training, cost of home visits by general practitioners, dietitians, nurses, speech and language therapists, physiotherapists, chiropodists, community occupational therapists, the cost of feeding, ancillaries and delivery, hospital readmissions and nursing home (b) No (c) No	United Kingdom 1995–2005 Pounds Sterling (2005)
**Beavan, J.;** **2010 [45]**	Data collected during randomised controlled trial, local purchase costs and the cost for a single loop set	(a) Yes, direct medical costs of feeding (b) No (c) No	United Kingdom 2006–2007 Pounds Sterling (year NA)
**Compensatory Strategies: Food Consistency Modification and Thickened Fluids**			
**Kotecki, S.;** **2010 [42]**	Data collected during study performance from a neuroscience hospitalisation unit	(a) Yes, direct medical costs of liquid products, thickeners, nurses and technicians, and viscometer (b) No (c) No	United States NA USD (year not available)
**Pelczarska, A.;** **2020 [43]**	Literature review Clinical expert consultations	(a) Yes, OD treatment costs, aspiration pneumonia treatment costs and monitoring costs (monitoring costs only for the dynamic model) (b) No (c) No	Poland NA PLN (year NA)

Abbreviations: ID: identification; NA: not available/not applicable; OD: oropharyngeal dysphagia; PLN: polish zloty; USD: United States dollars; VFS: videofluoroscopy.

**Table 3:** Specific characteristics of study populations.

Study ID	Age (Years) and Gender (Male)	Patient Inclusion or Exclusion Criteria	Method of OD Diagnostic
**Assessment of OD after Stroke**			
**Wilson, R.D.;** **2012** [38]	NA (model-based)	NA (model-based, typical hospitalised stroke patient without previous OD nor contraindication to OD screening)	VFS CBSE CBSE followed by VFS if abnormal swallow
**Svendsen, M.L.;** **2014** [37]	Mean age (SD): Processes received 0–24%: 72.2 (14.3) Processes received 25–49%: 73.6 (12.7) Processes received 50–74%: 72.6 (12.9) Processes received 75–100%: 69.9 (13.3) Gender *n* (%): Processes received 0–24%: 212 (47.9) Processes received 25–49%: 344 (49.9) Processes received 50–74%: 798 (54.1) Processes received 75–100%: 1875 (56.8)	Inclusion criteria: - ≥18 years - Hospitalised with acute stroke - First-time registered in the Danish Stroke Registry - Discharged from a stroke unit during the study period Exclusion criteria: - Patients hospitalised for more than one year	GUS
**Schwarz, M.;** **2017** [39]	Mean age (range): Overall: 69.9 (31–92) Gender *n* (%): Overall: 37 (44.6)	Inclusion criteria: - >18 years - Ischemic stroke Exclusion criteria: - Haemorrhagic stroke - Transient ischemic attack - Transferred to another facility or considered for palliative care during emergency department admission	Speech-language pathologist assessment Trained nursing staff using the Royal Brisbane and Women’s Hospital Dysphagia Screening Tool
**Liu, Z.Y.;** **2020** [36]	Mean age (range): Pre-V-VST: 69.73 (80.92–80.54) V-VST period: 67.36 (56.48–78.24) Gender *n* (%): Pre-V-VST: 93 (63.3) V-VST period: 55 (57.9)	Inclusion criteria: - ≥18 years - Ischemic stroke Exclusion criteria: - Tracheal intubation - Glasgow Coma Scale score of < 10 - More than 14 days after stroke onset passed	WST or WST followed by V-VST if the first failed
**Rehabilitation Services Including OD Management**			
**Khiaocharoen, O.;** **2012 [40]**	Mean age (SD): Control group: 60.8 (12.9) Rehabilitation group: 61.1 (12.5) Gender *n* (%): Control group: 53 (58.9) Rehabilitation group: 67 (57.3)	Inclusion criteria: - First stroke episode within 2 weeks after the onset - No other acute medical conditions requiring treatment - Without previous disability Exclusion criteria: - Bilateral hemiplegia or brain stem pathology - Depression - Barthel index score > 19 out of 20 - Surgery for stroke - Death - Critically ill at subacute and non-acute phases	NA
**Suksathien, R.;** **2015 [41]**	Mean age (SD): 57 (19–86) Gender *n* (%): 28 (56)	Inclusion criteria: - <18 years - Able to follow one-step command - Need rehabilitation - Had stable vital signs - Started the program not later than six months after stroke Exclusion criteria: - Patients who had serious complications - Cognitive impairments - Other causes that prevented patients from cooperating with rehabilitation program	NA
**Nutrition by Enteral Tube Feeding**			
**Elia, M.;** **2008 [44]**	Mean age (SD): Own home: 73 (13) Nursing home: 78 (10) Gender: NA	Inclusion criteria: - After cerebrovascular accident - Receiving long-term ETF in own homes or nursing homes	NA
**Beavan, J.;** **2010 [45]**	Mean age (SD): Loop group: 79 (10) Control group: 81 (10) Gender *n* (%): Loop group: 20 (39) Control group: 23 (43)	Inclusion criteria: - Acute stroke - NGT feeding required - Dysphagia identified Exclusion criteria: - Contraindications to NGT feeding - NGT feeding had been established for more than 7 days elsewhere	Standardised WST
**Compensatory Strategies: Food Consistency Modification and Thickened Fluids**			
**Kotecki, S.;** **2010 [42]**	NA	NA	NA
**Pelczarska, A.;** **2020 [43]**	NA (model-based)	NA (model-based, adult stroke patient with OD, analysis restricted to patients with an aspiration level of 10–14 on GUS)	GUS (model-based)

Abbreviations: CBSE: clinical bedside swallowing evaluation; EFT: enteral tube feeding; GUS: Gugging Swallowing Screen; ID: identification; NA: not available/not applicable; NGT: nasogastric tube; OD: oropharyngeal dysphagia; VFS: videofluoroscopy; V-VST: volume-viscosity swallow test; WST: water swallowing test.

**Table 4:** Results of individual studies and quality assessment.

Study ID	Aim	Sample Size	Specific Data Depending on the Type of Economic Evaluation	Result of Study: Cost Difference (+ Increase and − Reduction)/Incremental Cost-Effectiveness/Utility Ratio	Main Findings of Studies	Quality Assessment ^c^ (%)
**Assessment of OD after Stroke**						
**Wilson, R.D.;** **2012** [38]	Cost-effectiveness of dysphagia screening methods	NA	Cost: VFS: 1853 USD CBSE: 1968 USD CBSE plus VFS: 1943 USD QALY: VFS: 1.791 QALYs CBSE: 1.789 QALYs CBSE plus VFS: 1.790 QALYs	Incremental effectiveness: VFS: NA CBSE plus VFS (vs. VFS): −0.001 CBSE (vs. CBSE plus VFS): −0.001 Incremental costs: VFS: NA CBSE plus VFS (vs. VFS): + 90 USD CBSE (vs. CBSE plus VFS): + 25 USD Cost/QALY of VFS: 1034 USD	VFS was the most cost-effective screening method compared to CBSE and a combination of both	82.1
**Svendsen, M.L.;** **2014** [37]	Association between processes of early stroke care and hospital costs	5909	Mean crude costs: Early swallowing assessment (first 24 h): 20,232 (25,459) ^a^ USD Delayed swallowing assessment: 29,222 (30,177) USD Mean adjusted costs: Early swallowing assessment (first 24 h): 19,487 (10,662) ^a^ USD Delayed swallowing assessment: 32,043 (15,097) USD	Adjusted cost difference: −12,556 (95% CI 9751–15,361) USD	Reduction in hospitalisation costs of 12,556 USD when swallow was assessed during the first admission day	78.8
**Schwarz, M.;** **2017** [39]	Impact of an OD screening protocol for thrombolysed stroke patients on operational outcomes (cost, length of stay, service compliance)	83	Costs: Screening protocol: 16,548 Australian dollars Non screening protocol: 18,053 Australian dollars	Crude costs difference: −1505 Australian dollars (*p* = 0.722; F = 0.129)	Non-significant reduction of 1505 Australian dollars in hospitalisation costs using a protocol to manage OD after thrombolysis	61.5
**Liu, Z.Y.;** **2020** [36]	Differences in total hospitalisation costs assessing OD with the WST test vs. the WST test and the V-VST if the first failed	242	No differences in median total hospitalisation costs: -WST group: 2807.8 (1951.4–4461.5) ^b^ -WST followed by V-VST: 2899.4 (2012.9–5074.7) ^b^ *p* = 0.0846	NA	No differences in hospitalisation costs when PS-OD was assessed with the WST vs. V-VST if the WST failed	57.6
**Rehabilitation Services Including OD Management**						
**Khiaocharoen, O.;** **2012 [40]**	Cost-utility of rehabilitation programme including swallow training	207	Incremental program costs: - Hospital perspective: 5592 bahts - Societal perspective: 6880 bahts QALY: Rehabilitation group: 0.632 Control group: 0.352	ICUR (Cost/QALY): - Governmental perspective: 19,971 bahts - Societal perspective: 24,571 bahts	ICUR of rehabilitation programme including swallow training (starting at the subacute and non-acute stroke phases) of 24,571 bahts from societal perspective	79.6
**Suksathien, R.;** **2015 [41]**	Efficiency and cost of short-course rehabilitation program	50	Change in BI score between admission and discharge: 5.00 (2.25) ^a^ Total cost of rehabilitation admission: 7729 (4330) ^a^ 95% CI 1828–22,450 bahts	Change score of the BI/LOS: 0.56 (0.33) Cost/change score of the BI: 1545.8 bahts	Positive mean change in the BI score of 5 points between discharge and admission with mean total costs of 7729 bahts	54.1
**Nutrition by Enteral Tube Feeding**						
**Elia, M.;** **2008 [44]**	Cost-utility of long-term enteral tube feeding	9895 QoL assessment (*n* = 25)	QoL (EuroQol): Home: 0.47 (0.28) ^a^ Nursing home: 0.47 (0.25) ^a^ QoL (both groups): 0.47 (95% CI 0.358–0.582) Mortality at 2 years: Home: 43% Nursing home: 56%	ICUR (Cost/QALY): - Patients at home: £12,817 (95% CI for quality of life £10,351–£16,826) - Nursing home: £10,303–£68,064	ICUR of home enteral nutrition of £12,817 ICUR of nursing home enteral nutrition of £10,303–£68,064	81.4
**Beavan, J.;** **2010 [45]**	Cost-effectiveness of nasal loop	104	Percentage of received nutrition of total prescribed: - Looped nasogastric tube: 75% (95% CI 67–83%) - Control group: 57% (95% CI 49–65%) Incremental percentage: 17% (95% CI 5–28%) Mean feeding costs: - - Looped nasogastric tube: £426 - - Control group: £338	Incremental cost for an 1% additional total nutrition received: + £5.20	Higher nutrient intake and low increase in hospitalisation costs using looped-nasogastric tube (£5.20 for every 1% increase)	70.3
**Compensatory Strategies: Food Consistency Modification and Thickened Fluids**						
**Kotecki, S.;** **2010 [42]**	Differences in time and costs between nursing-staff-prepared and commercially prepared thickened liquids	NA	Cost of preparing thickened liquids: Nectar texture: Water: 0.54 USD Milk: 1.34 USD Orange Juice: 0.86 USD Honey texture: Water: 0.75 USD Milk: 1.41 USD Orange Juice: 0.83 USD Commercially prepared products: Nectar texture: Water (4 ounces): 0.30 USD Milk (8 ounces): 0.61 USD Orange Juice (4 ounces): 0.36 USD Honey texture: Water (4 ounces): 0.31 USD Milk (8 ounces): 0.66 USD Orange Juice (4 ounces): 0.36 USD	Cost savings using commercially prepared thickened liquids: Nectar texture: Water: 44% Milk: 54% Orange Juice: 58% Honey texture: Water: 59% Milk: 53% Orange Juice: 57%	Commercially prepared thickened fluids 44% to 59% cheaper than in situ prepared	71.7
**Pelczarska, A.;** **2020 [43]**	Cost-utility of xanthan gum-based consistency modification therapy (Nutilis Clear^®^)	NA	QALY and total costs: Static model: - Nutilis Clear ^®^: 0.057 QALYs, total cost of 970 PLN - RCP: 0.022 QALYs, total cost of 235 PLN Dynamic model: - Nutilis Clear ^®^: 0.351 QALYs, total cost of 994 PLN - RCP: 0.331 QALYs, total cost of 570 PLN	ICUR (Cost/QALY): Static model: 21,387 PLN Dynamic model: 20,977 PLN	ICUR of texture-modified diets using a gum-based thickener of 20,977 PLN following a dynamic model and of 21,387 PLN following a static model	83.9

Abbreviations: BI: Barthel index; ICUR: incremental cost–utility ratio; ID: identification; LOS: length of hospital stays; PLN: polish zloty; QALY: quality-adjusted life-year; QoL: quality of life; RCP: routine clinical practice; USD: United States dollars. ^a^ values are mean (SD); ^b^ values are median (interquartile range); ^c^ Quality assessment: a higher score indicates a lower risk of bias. Score calculation: [Yes (1) + Partly (0.5)/Total applicable] × 100.

## Data Availability

No additional data is available.

## Supplementary Materials

The following supporting information can be downloaded at: https://www.mdpi.com/article/10.3390/nu15071714/s1, Table S1: Quality assessment adapted from the Consolidated Health Economic Evaluation Reporting Standards 2022 (CHEERS 2022) Statement Checklist and Literature search section, describing full search terms used in the bibliographic search of the four databases.
