# Supplementary material for: Economic Evaluation of Clinical, Nutritional and Rehabilitation Interventions on Oropharyngeal Dysphagia after Stroke: A Systematic Review
Source: Nutrients. 2023 Mar 31;15(7):1714. doi: 10.3390/nu15071714 (PMC10097035; doi:10.3390/nu15071714)
Supplement: Supplementary file 1 [file nutrients-15-01714-s001.zip › nutrients-2277754-supplementary.pdf]

Supplementary material

JOURNAL

Nutrients

TITLE

Economic evaluation of clinical, nutritional and rehabilitation interventions on oropharyngeal dysphagia after stroke: a systematic review

AUTHORS

1. Marin, Sergio. Academic degree: Pharmacist. Affiliations: 1. Gastrointestinal Physiology Laboratory, Hospital de Mataró, Universitat Autònoma de Barcelona, Mataró, Catalunya, ES. 2. Pharmacy Department, Hospital Universitari Germans Trias i Pujol, Badalona, Catalunya, ES

2. Ortega, Omar. Academic degree: Biologist, PhD in Medicine. Affiliations: 1. Gastrointestinal Physiology Laboratory, Hospital de Mataró, Universitat Autònoma de Barcelona, Mataró, Catalunya, ES. 2. Centro de Investigación Biomédica en Red de enfermedades hepáticas y digestivas (CIBERehd), Instituto de Salud Carlos III, Barcelona, Catalunya, ES.

3. Serra-Prat, Mateu. Academic degree: Medical Doctor, PhD in Medicine. Affiliations: 1. Research Unit, Consorci Sanitari del Maresme, Mataró, Catalunya, ES. 2. Centro de Investigación Biomédica en Red de enfermedades hepáticas y digestivas (CIBERehd), Instituto de Salud Carlos III, Barcelona, Catalunya, ES.

4. Valls, Ester. Academic degree: Pharmacist. Affiliations: 1. Pharmacy Department, Hospital Universitari Germans Trias i Pujol, Badalona, Catalunya, ES.

5. Pérez-Cordón, Laia. Academic degree: Pharmacist. Affiliations: 1. Pharmacy Department, Consorci Sanitari del Maresme, Hospital de Mataró, Mataró, Catalunya, ES.

6. Clavé, Pere. Academic degree: Professor, Medical Doctor, PhD in Medicine. Affiliations:1. Gastrointestinal Physiology Laboratory, Hospital de Mataró, Universitat Autònoma de Barcelona, Mataró, Catalunya, ES. 2. Centro de Investigación Biomédica en Red de enfermedades hepáticas y digestivas (CIBERehd), Instituto de Salud Carlos III, Barcelona Catalunya, ES.

Literature search

Search terms and MeSH terms used in the bibliographical search in MEDLINE using PubMed.

(“Deglutition”[MeSH] OR “Deglutition Disorders”[MeSH] OR “Oropharynx/abnormalities”[MeSH] OR “Oropharynx/diagnosis”[MeSH] OR “Oropharynx/diagnostic imaging”[MeSH] OR “Oropharynx/pathology”[MeSH] OR “Oropharynx/pharmacology”[MeSH] OR “Oropharynx/physiopathology”[MeSH] OR “Oropharynx/therapy”[MeSH] OR Enteral tube feed*/ OR Swallow[tiab] OR Dysphag*[tiab] OR Deglut*[tiab] OR Dysphagia[tw] OR Dysphag*/ OR Dysphagia therapy/) AND (“Stroke”[MeSH] OR “Stroke Rehabilitation”[MeSH] OR “Brain Ischemia/ complications”[MeSH] OR “Cerebral Infarction”[MeSH] OR “Cerebral Hemorrhage”[MeSH] OR “Intracranial Embolism and Thrombosis”[MeSH] OR “Intracranial Hemorrhages”[MeSH] OR “Intracranial Arteriosclerosis”[MeSH] OR “Cerebrovascular Disorders”[MeSH] OR Stroke[tiab] OR Post stroke[tiab] OR Poststroke[tiab] OR Post-stroke[tiab] OR Cerebral Ischaemia[tiab] OR Brain Ischaemia[tiab] OR Brain infarction[tiab] OR Intracranial hemorrhage[tiab] OR Intracranial haemorrhage[tiab] OR Cerebral Hemorrhage[tiab] OR Cerebral Haemorrhage[tiab] OR Brain Hemorrhage[tiab] OR Brain Haemorrhage[tiab] OR Stroke discharge/ OR Post-stroke/) AND (“Economics”[MeSH] OR “Economics”[Subheading] OR “Models, Economic”[MeSH] OR “Health Resources”[MeSH] OR “Tertiary Care Centers/economics”[MeSH] OR “Rehabilitation Centers/economics”[MeSH] OR “Lenght of Stay/economics”[MeSH] OR “Medicare/economics”[MeSH] OR “Physical Therapy Modalities/economics”[MeSH] OR “Emergency Medical Services/economics”[MeSH] OR “Food, Formulated/economics”[MeSH] OR “Cerebrovascular Disorders/economics”[MeSH] OR Cost effectiveness analysis OR Cost utility analysis OR Cost minimization analysis OR Cost benefit analysis OR Cost[tw] OR Costs[tw] OR Quality-adjusted life years/ OR Cost utility[tiab] OR Cost-utility[tiab] OR Cost benefit[tiab] OR Cost-benefit[tiab] OR Cost minimization[tiab] OR Cost-minimization[tiab] OR Cost effeciveness[tiab] OR Cost-effectiveness[tiab])

Search terms used in the bibliographical search in Embase using Ovid.

(“Deglutition” OR “Deglutition Disorders” OR “Dysphagia”) AND (“Stroke” OR “Post stroke” OR “Poststroke” OR “Post-stroke” OR “Cerebral Ischaemia” OR “Brain Ischaemia” OR “Brain infarction“ OR “Cerebral Hemorrhage” OR “Cerebral Haemorrhage” OR “Brain Hemorrhage” OR “Brain Haemorrhage”) AND (“Economics” OR “Cost utility” OR “Cost minimization” OR “Cost benefit” OR “Cost” OR “Costs” OR “Cost-effectiveness”)

Search terms used in the bibliographical search in the National Health Service Economic Evaluation Database (NHS-EED) using the Center for Reviews and Dissemination Database of the University of York.

((Dysphagia OR Deglutition Disorders OR Enteral tube feeding OR Dysphagia therapy OR Oropharynx abnormalities OR Oropharynx diagnosis OR Oropharynx diagnostic imaging OR Oropharynx pathology OR Oropharynx pharmacology OR Oropharynx physiopathology OR Oropharynx therapy)) and ((Economic evaluation:ZDT and Bibliographic:ZPS) OR (Economic evaluation:ZDT and Abstract:ZPS)) IN NHSEED

Search terms used in the bibliographical search in the Cost-Effectiveness Analysis (CEA) Registry database of the Center for the Evaluation of Value and Risk in Health.

(Dysphagia OR Deglutition Disorders OR Enteral tube feeding OR Dysphagia therapy OR Oropharynx abnormalities OR Oropharynx diagnosis OR Oropharynx diagnostic imaging OR Oropharynx pathology OR Oropharynx pharmacology OR Oropharynx physiopathology OR Oropharynx therapy)

No publication date and no language restrictions were imposed.
Unpublished material, protocols, books and abstracts were not included in this systematic review.
No other filters or limits were imposed.

Reference: Marin, S.; Serra-Prat, M.; Ortega, O.; Clavé, P. Economic evaluations of health care interventions in Oropharyngeal Dysphagia after Stroke: Protocol for a Systematic Review. *Syst Rev.* **2022**, 11, 92. doi: 10.1186/s13643-022-01969-6.

Table S1: Quality assessment adapted from the Consolidated Health Economic Evaluation Reporting Standards 2022 (CHEERS 2022) Statement Checklist

**Section/topic**	**Elia M 2008**	**Beavan J 2010**	**Kotecki S 2010**	**Khiaocharoen 2012**	**Wilson RD 2012**	**Svendsen ML 2014**	**Suksathien R 2015**	**Schwarz M 2017**	**Liu ZY 2020**	**Pelczarska A 2020**
TITLE										
1 Title	Yes	No	Yes	Yes	Yes	Yes	No	No	No	Yes
ABSTRACT										
2 Abstract	Partly	Partly	Yes	Yes	Yes	Yes	Yes	Partly	Partly	Yes
INTRODUCTION										
3 Background and objectives	Yes	Yes	Yes	Yes	Yes	Yes	Yes	Yes	Partly	Yes
METHODS										
4 Health economic analysis plan	No	No	No	No	No	No	No	No	No	No
5 Study population	Yes	Yes	Yes	Yes	Yes	Yes	Partly	Yes	Yes	Yes
6 Setting and location	Yes	Yes	Yes	Yes	Yes	Yes	Yes	Yes	Yes	Yes
7 Comparators	Yes	Yes	Yes	Yes	Yes	Yes	NA	Yes	Yes	Yes
8 Study perspective	Partly	Yes	Yes	Yes	Yes	Yes	No	Partly	Yes	Yes
9 Time horizon	Yes	Yes	Yes	Yes	Yes	Partly	Yes	Yes	Yes	Yes
10 Discount rate	Yes	No	NA	No	NA	NA	NA	NA	NA	NA
11 Selection of outcomes	Yes	Yes	Yes	Yes	Yes	Partly	Yes	Partly	Partly	Yes
12 Measurement of outcomes	Yes	Yes	Yes	Yes	Yes	Yes	Yes	Partly	Yes	Yes
13 Valuation of outcomes	Yes	Yes	Yes	Yes	Yes	Yes	Yes	Yes	Yes	Yes
14 Measurement and valuation of resources and costs	Yes	Yes	Yes	Yes	Yes	Yes	Partly	Partly	Partly	Yes
15 Currency, price date, and conversion	Yes	Partly	Partly	Partly	Yes	Yes	Partly	Partly	Partly	Partly
16 Rationale and description of model	NA	NA	NA	NA	Yes	NA	NA	NA	NA	Yes
17 Analytics and assumptions	Yes	Yes	Partly	Yes	Yes	Yes	Partly	Partly	Partly	Yes
18 Characterising heterogeneity	Yes	Yes	NA	Yes	Yes	Yes	NA	Partly	Partly	Yes
19 Characterising distributional effects	Yes	No	NA	No	Yes	Partly	No	No	No	Yes
20 Characterising uncertainty	Yes	Partly	No	Yes	Yes	Partly	No	Partly	No	Yes
21 Approach to engagement with patients and others affected by the study	No	No	No	No	No	No	No	No	No	No
RESULTS										
22 Study parameters	Yes	Yes	Yes	Yes	Yes	Yes	Yes	Yes	Yes	Yes
23 Summary of main results	Yes	Yes	Yes	Yes	Yes	Yes	Yes	Yes	Partly	Yes
24 Effect of uncertainty	Yes	Partly	No	Yes	Yes	Partly	No	Partly	No	Yes
25 Effect of engagement with patients and others affected by the study	No	No	NA	No	No	No	No	No	No	No
DISCUSSION										
26 Discussion	Yes	Yes	Partly	Yes	Yes	Yes	Yes	Yes	Yes	Yes
OTHER RELEVANT INFORMATION										
27 Source of funding	No	Yes	Partly	Yes	Yes	Yes	No	Yes	Yes	Yes
28 Conflicts of interest	Yes	Yes	Partly	Yes	No	Yes	Yes	Yes	Yes	Yes
